# Peri-Implant Diseases: Diagnosis, Clinical, Histological, Microbiological Characteristics and Treatment Strategies. A Narrative Review

**DOI:** 10.3390/antibiotics9110835

**Published:** 2020-11-22

**Authors:** Ioannis Kormas, Chantal Pedercini, Alessandro Pedercini, Michail Raptopoulos, Hatem Alassy, Larry F. Wolff

**Affiliations:** 1 Division of Periodontology, Department of Developmental and Surgical Sciences, School of Dentistry, University of Minnesota, Minneapolis, MN 55455, USA; pede0783@umn.edu (A.P.); rapto003@umn.edu (M.R.); alassy@umn.edu (H.A.); wolff001@umn.edu (L.F.W.); 2 School of Oral Surgery, Vita-Salute San Raffaele University, 20132 Milan, Italy; c.pedercini@studenti.unisr.it

**Keywords:** peri-implantitis, peri-implant mucositis, peri-implant disease, risk factors, antibiotics

## Abstract

Since the use of dental implants is continuously increasing, it is imperative for dental practitioners to understand the nature and treatment of peri-implant diseases. The purpose of this manuscript is to comprehensively review peri-implant diseases, their characteristics, as well as their non-surgical and surgical treatment. To that end, the current literature was searched and a narrative review was conducted. It is essential that the case definitions described in the 2017 World Workshop on the Classification of Periodontal and Peri-implant Diseases and Conditions are used to diagnose and classify peri-implant health, peri-implant mucositis and peri-implantitis. While recent epidemiologic studies on peri-implant diseases exist, there is great heterogeneity in the definition of these conditions. Several risk factors and indicators are reported in the literature, with smoking and diabetes being the most universally accepted. In peri-implant mucositis, non-surgical treatment seems to be sufficient. However, for the treatment of peri-implantitis, a surgical approach, which includes open-flap debridement, apically positioned flap and guided bone regeneration, is considered more appropriate. A great variety of adjuncts to mechanical treatment have been reported with controversial results. Finally, studies comparing results from different peri-implantitis treatments are warranted in randomized controlled clinical trials in order to provide stronger evidence-based approaches.

## 1. Introduction

The worldwide prevalence of edentulism was estimated from a systematic review and meta-analysis at 4.4% in 1990 and it decreased to 2.4% in 2010 [1]. Dental implants have contributed to the reduction of the edentulous patients globally. Treatment of edentulism with dental implants is a routine treatment in daily dental practice and is becoming more widespread among practitioners as the years progress.

From a cross-sectional study on the Swiss population, the percentage of dentists that are engaged in implant dentistry doubled in 12 years, from 42.2% in 1994 to 82.2% in 2006, while an additional 10% increase occurred in the next 10 years [2,3]. In the United States, the 1999–2000 prevalence of dental implants was 0.7%, but increased to 5.7% in 2015–2016 [4]. The increase in the dental implants’ prevalence is currently 14% per year and is projected to reach up to 23% in the year 2026. Dental implants are reliable treatments with a mean survival rate of 94.6% [5]. Particularly, implants with the use of the commercially pure titanium as well as with the Ti-6Al-4V alloy can reach survival rates of up to 99% [6]. The currently well-established surface processing with sandblasting and acid etching allowed for shorter osseointegration time and increased bone-implant contact [7]. However, with the increasing use of dental implants, peri-implant diseases are also becoming more prevalent and their clear definition and understanding of their treatment is essential. The purpose of this manuscript is to present a comprehensive review on peri-implant diseases, particularly focusing on case definitions, characteristics and treatment strategies. Several publications exist on the subject of peri-implant conditions, but this review can serve as a guide to the clinicians and surgeons on the latest treatments and approaches to manage and treat peri-implantitis.

## 2. Criteria for Implant Success

Over the years, multiple studies have defined the criteria for implant success. While other classifications of implant success preceded it, the classification of Albrektsson et al. in 1986 has been the baseline for most recent investigations of implant success [8]. The described criteria were absence of mobility, no evidence of radiolucency around the implant, <0.2 mm of annual bone loss after the first year of function, lack of persistent and/or irreversible signs and symptoms such as pain, infections, neuropathies, paresthesia, or violation of the mandibular canal. Buser and colleagues in 1990 proposed the following criteria of success: (1) absence of persistent symptoms such as pain, sensation of foreign body and/or dysesthesia, (2) absence of recurrent peri-implant infection with suppuration, (3) no mobility, (4) absence of peri-implant radiolucency and (5) feasibility of restoration [9]. In 2008, at the International Congress of Oral Implantologists (ICOI) Pisa Consensus Conference, the implant success, survival and failure were redefined [10]. Implant success was defined as no pain or tenderness upon function, absence of mobility, <2 mm radiographic bone loss from the initial surgery and no presence of exudate.

In a systematic review, a more comprehensive evaluation of the criteria of implant success was reported at different levels: implant, peri-implant soft tissue, prosthetic and patient [11]. At the implant level, criteria for success were absence of mobility, pain, radiolucency and peri-implant bone loss (>1.5 mm at 1st year). When the level of the peri-implant soft-tissue is assessed, criteria for success should be absence of suppuration and bleeding. Implant success at the prosthetic level includes lack of technical/prosthetic complications, adequate function and esthetics. At the patient level, the criteria for success were satisfaction with esthetics and ability to chew/taste without any discomfort and/or paresthesia.

## 3. Peri-Implant Health and Diseases: Case Definitions

**Figure 1:**
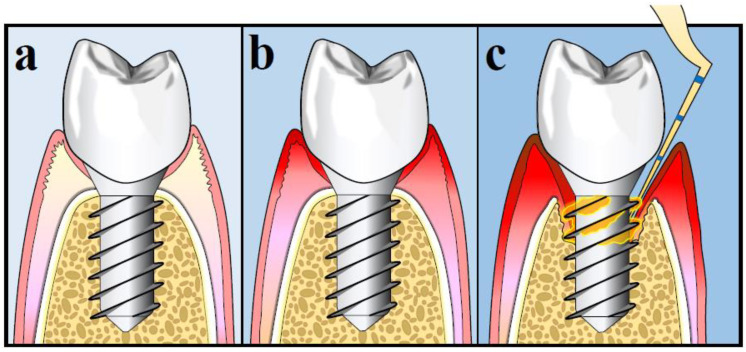
Peri-implant health/disease status: (**a**) Peri-implant health; (**b**) Peri-implant mucositis; (**c**) Peri-implantitis.

The 2017 World Workshop addressing the Classification of Periodontal and Peri-implant Diseases and Conditions provided specific criteria to accurately define peri-implant status which are summarized in Table 1 [12]. Figure 1 depicts the peri-implant health/disease status. Peri-implant health is defined as absence of peri-implant signs of soft tissue inflammation, absence of bleeding and/or suppuration upon gentle probing, no increase in probing depth (PD) compared to previous visits and absence of radiographic bone loss (RBL) beyond the crestal bone level changes that occurred due to initial bone remodeling after implant placement [12,13,14]. Peri-implant mucositis is characterized by the presence of bleeding and/or suppuration upon gentle probing with or without increased PD compared to previous examinations and also absence of additional RBL changes that occurred after the initial remodeling of the bone [12,14,15].

The definition of peri-implantitis depends on the presence or absence of previous records. Utilizing existing previous records, peri-implantitis is defined as a presence of peri-implant signs of bleeding and/or suppuration upon gentle probing, increased PD compared to previous examinations and presence of RBL beyond crestal bone level changes after initial bone remodeling, which should not be ≥2 mm. However, in the absence of previous radiographic records, the signs used for case definition of peri-implantitis are the presence of bleeding and/or suppuration upon gentle probing, PD ≥6 mm and RBL ≥3 mm apical to the most coronal part of the intraosseous portion of the implant [12,14,16].

Furthermore, there are various hard and soft tissue deficiencies in relation to implants. An effort to classify and describe the etiology of these hard and soft tissue deficiencies such as but not limited to ridge deficiency, recession, lack of keratinized tissue (KT) and inadequate mucosal thickness was made at the 2017 World Workshop on the Classification of Periodontal and Peri-implant Diseases and Conditions [17].

## 4. Epidemiology of Peri-Implant Diseases

There have been multiple epidemiological studies that investigated the prevalence of peri-implant diseases. In a systematic review and meta-analysis conducted in 2015 of 11 epidemiological studies, the prevalence of peri-implant mucositis in the studies evaluated ranged from 19 to 65% and peri-implantitis ranged from 1 to 47% [18]. In another systematic review and meta-analysis performed in 2017, from a total of 47 studies, the mean average for peri-implant mucositis at the implant level was 29.48%, while on a patient level it was 46.83% [19]. On the other hand, in this same systematic review, the mean averages at the implant and patient levels were 9.25% and 19.83%, respectively. The prevalence for peri-implantitis from a systematic review of 29 studies in 2018 were 12.8% (implant level) and 18.5% (patient level) [20]. Another recent systematic review and meta-analysis including 57 articles reported a wider range for implant-level peri-implantitis’ prevalence ranging from 1.1% to 85% among the studies [21]. This same review also reported on the incidence of peri-implantitis, ranging from 0.4% over 3 years, to 43.9% within 5 years after implant restoration. While those results are limited by the heterogeneity of the included studies, they reflect the high prevalence of peri-implant diseases. The heterogeneity of the studies exists primarily due to the absence of specific criteria for the definition of peri-implantitis and its clinical features. In 2019, a new review on the prevalence of peri-implantitis was conducted taking into consideration the case definition of the 2017 Workshop for the Classification of Periodontal and Peri-implant Diseases and Conditions [12,22]. The authors concluded that it was important for new cross-sectional and epidemiological studies to be conducted using the universally accepted new criteria for the classification of peri-implant diseases.

## 5. Risk Factors and Risk Indicators for Peri-Implant Diseases

**Figure 2:**
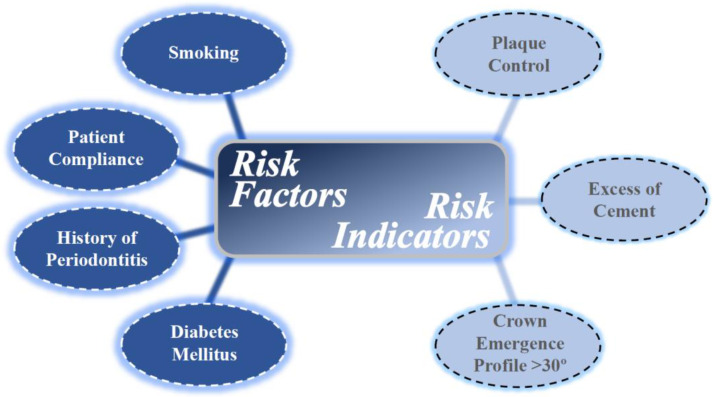
Risk factors and indicators of peri-implant diseases.

Risk factors are causal agents of a disease which are usually confirmed by longitudinal studies. On the other hand, risk indicators are based on cross-sectional data [23]. A large number of risk factors and indicators have been associated with peri-implant diseases and are represented in Figure 2. The most well-established systemic risk factors that have been consistently associated with peri-implant diseases are smoking and diabetes mellitus.

In a recent systematic review and meta-analysis, smoking was found to present a significantly higher risk for peri-implantitis on an implant level (RR: 2.1, 95% CI: 1.34–3.29, *p* = 0.001) when compared to non-smokers. However, at a patient level, no significant association was identified (RR: 1.17, 95% CI: 0.78–1.75, *p* = 0.46) [24]. The significance of smoking as a risk factor for the development of peri-implantitis, as well as peri-implant mucositis, has also been reported in several other studies [21,25,26,27,28,29,30]. Diabetes has also been associated with peri-implantitis as a significant risk factor, demonstrating twice the risk for developing peri-implantitis [21,31,32,33,34]. Diabetes mellitus is a pro-inflammatory systemic condition with altered immune response affecting both catabolic and anabolic events of bone-healing that include increased osteoclastogenesis and compromised osteoblast activity [34]. Furthermore, chronic hyperglycemia along with associated micro- and macro-vascular disorders may result in delayed and/or impaired wound healing due to the activation of pathways linked to inflammation, oxidative stress, and cell apoptosis. A higher risk of peri-implantitis was detected in people with hyperglycemia [33]. Meta-analyses also estimated the risk of peri-implantitis at 50% greater in diabetics compared to non-diabetic subjects (RR: 1.46, 95% CI: 1.21–1.77, *p* < 0.001) [32].

Patient compliance is also an important risk factor for maintaining implant health. The lack of regular prophylaxis resulted in a tendency for higher risk of developing peri-implantitis [21]. Additionally, presence or history of periodontitis has been shown to pose a significant risk for developing peri-implantitis compared to patients who do not have periodontitis [21,35,36]. With respect to patient oral hygiene, a plaque control record score of >20% (hazard ratio = 2.61) is considered a significant risk indicator for peri-implantitis [37,38]. Moreover, an emergence profile of the implant restoration exceeding 30 degrees has been identified as a significant risk indicator for peri-implantitis in bone-level implants, especially if there is a convex crown profile [39]. In cementable restorations, excess cement has also been demonstrated to be a risk indicator for peri-implant disease [40,41,42,43].

Multiple other parameters have been reported as risk factors or indicators for peri-implantitis and peri-implant mucositis by several studies, but with contradictory results. Among these risk factors or indicators associated with peri-implantitis or peri-implant mucositis, the most commonly encountered have been age, gender, osteoporosis, implant location, implant surface characteristics and bone augmentation of the site prior to implant placement [21,27,38,44]. Additional studies are needed to clarify these parameters as risk factors or indicators for peri-implant diseases. One of the most debated factors for peri-implant diseases is the presence/absence of KT around the implant. The heterogeneity of the results in the literature does not allow for a valid, evidence-based conclusion [21]. The current consensus is that, even if the presence of KT cannot be universally supported by the literature, the presence of 2 mm of KT is desired [45].

## 6. Implant Prognosis

While a number of models have been reported on the prognosis of natural teeth, there are very few prognostication systems for implants. Using data from a survey on prosthodontists, periodontists and oral surgeons, a system divided the prognosis of the implants into good, questionable, poor and hopeless [46]. The criteria used for the prognosis in the study were evaluated at implant, peri-implant soft tissue, prosthetic and patient levels. However, the reliability of a prognostication system on a blind survey is low. A prognostic model was developed, based on retrospective 1-year results of Nobel Biocare implants [47]. The algorithm took into consideration factors that were found to be significant: age, history of periodontitis, severe peri-implant disease status, implant length and early disease development. However, this model was based on retrospective data and a follow-up of only one year is a short-term model. There is a need for systems of prognosis on implants based on prospective longitudinal studies, and thus far in the publications, there seems to be a lack thereof in the literature.

## 7. Clinical and Radiographic Analysis of Peri-Implantitis

Clinical signs of peri-implantitis reflect the previously described case definition according to the 2017 World Workshop on the Classification of Periodontal and Peri-Implant Diseases and Conditions [12]. Clinical signs of inflammation include erythema, edema, mucosal enlargement and bleeding on probing (BOP), with or without suppuration [16]. In peri-implantitis sites, these findings are accompanied by an increase in PD and RBL. A recent publication evaluated the clinical characteristics of peri-implantitis sites in comparison with peri-implant mucositis, as well as healthy implant sites [48]. Healthy implant sites were associated with an absence of BOP, while among all included implants, the calculated mean BOP at peri-implant mucositis and peri-implantitis sites were 43% and 86%, respectively. However, peri-implantitis sites demonstrated significantly greater PDs compared to peri-implant mucositis sites. In this same study, suppuration was limited to peri-implantitis patients.

When a diagnosis of peri-implantitis is made, the distinction between ailing, failing and a failed implant may be clinically relevant [49]. An ailing implant is characterized by RBL without clinical signs of inflammation; the previously present inflammation may have been halted and a deep PD is accompanied by absence of BOP, a status which needs monitoring. A failing implant, in addition to RBL, appears with signs of inflammation such as BOP, edema, redness and suppuration; clinical intervention may be successful in this case. A failed implant manifests with severe RBL associated with mobility; explantation is the treatment of choice in such case scenarios.

The radiographic assessment of peri-implantitis is generally conducted with periapical films. The mean RBL measured was found to be an underestimation of the intra-surgical bone defect [50]. Furthermore, the ability to take standardized radiographs from baseline to follow-up may be crucial for a reliable diagnosis [51]. Radiographic analysis of peri-implantitis has been evaluated retrospectively, and it appeared that peri-implantitis-associated bone loss is characterized by a non-linear progression which increases over time [52,53]. These findings were corroborated by a more recent study with a follow-up of 9 years [54]. Radiographs from 62 subjects who were diagnosed with peri-implantitis at ≥1 implant at their recall appointments were collected, and the marginal RBL was assessed over time and compared to baseline. It was observed the radiographic progression of peri-implantitis was not linear and it accelerated over time. In addition, almost 50% of the implants presented >1 mm of marginal RBL within 3 years of function. The pattern of RBL in peri-implantitis sites was vertical, horizontal and combined in 65%, 22% and 13% of the cases, respectively [44].

## 8. Histopathology and Microbiology of Peri-Implantitis

Several histopathologic animal studies have been conducted in order to clarify the difference in the pattern of progression between peri-implantitis and periodontitis [55,56,57,58]. Although clinical manifestations were quite similar, the RBL progression of peri-implantitis sites was significantly greater compared to the periodontitis ones. Furthermore, between the inflammatory cell infiltrate and the alveolar bone crest, non-inflammatory supra-alveolar connective tissue was found in periodontitis sites; whereas in peri-implantitis sites, the infiltrate occupied a much larger volume extending into the alveolar bone. These data have been verified by human studies, where a large inflammatory cell infiltrate was found in almost the entire peri-implant connective tissue portion [59,60]. The infiltrate was dominated by plasma cells, but unlike periodontitis sites, numerous polymorphonuclear leukocytes (PMN) were also present in peri-vascular compartments in more central areas of the inflammatory cell infiltrate. This might suggest an enhanced PMN cell activity at sites with peri-implantitis. Additionally, foreign bodies such as titanium and dental cement surrounded by inflammatory cells were revealed from human peri-implantitis biopsies [61]. However, the potential role of these foreign particles in the pathogenesis of peri-implantitis is still unknown. Histologically, the peri-implant mucosa was found to establish a cuff-like barrier which adhered to the surface of the titanium abutment. In contrast to the periodontium, the collagen fibers of the peri-implant mucosa were parallel to the abutment surface [62]. Although this feature seems to offer a weaker seal for the peri-implant soft tissue compared to the connective tissue attachment of natural teeth, it was concluded that both types of adherence have a proper potential to prevent subgingival plaque formation.

From a microbiological aspect, despite the similarities between the microbiota in healthy peri-implant tissue and healthy periodontium, the diseased implant surface may be colonized with pathogens different from periodontopathogenic bacteria found associated with periodontitis around natural teeth [63,64]. *Staphylococcus* spp., enterics and *Candida* spp. were revealed in 55% of the peri-implant lesions. From a microbiological comparison between healthy implant and peri-implantitis sites, 19 species were found in higher counts in peri-implantitis, demonstrating that a bacterial cluster may be associated with the onset of peri-implantitis [65]. Particularly, the bacterial counts of *Tannerella forsythia*, *Treponema denticola*, *Campylobacter rectus*, *Treponema socranskii*, *Porphyromonas gingivalis*, *Staphylococcus aureus*, *Campylobacter gracilis* and *Prevotella intermedia* had significantly greater levels in terms of odds ratios for subjects with peri-implantitis when compared to peri-implant health. *Candida* spp. and other fungal organisms were also frequently found in higher counts at peri-implantitis sites compared to healthy ones, further suggesting that these microflora counts may play a role in the onset of peri-implantitis [66]. A higher prevalence of human cytomegalovirus and Epstein–Barr virus has also been reported in subgingival plaque of peri-implantitis sites and may indicate potential pathogenic activity at peri-implantitis as well [67].

## 9. Biomarkers of Peri-Implantitis

During the active state of periodontitis, inflammatory cells, cytokines, proteins, proteinases and local tissue-degradation products are released into the gingival crevicular fluid (GCF), whose content could be an important source of biomarkers [68]. Great interest is now focused on potential biomarkers found also in peri-implant sulcus fluid (PISF), used interchangeably with peri-implant crevicular fluid (PICF), which could be of diagnostic value for peri-implant diseases [69]. Increased levels of collagenolytic matrix metalloproteinase 8 (MMP-8) and interleukin 1β (IL-1β) have shown to be associated with inflammation, and lower levels of matrix metalloproteinase 1 (MMP-1) and tissue inhibitor of MMP 1 (TIMP-1) may be indicators of disease progression around implants [70,71]. However, there is still limited evidence from controlled longitudinal data and further research in this field is warranted. Moreover, conducting a meta-analysis on this topic has been very challenging due to the heterogeneity of the studies [72]. Regarding micro-RNA expression in peri-implantitis, there is very limited evidence on their diagnostic and prognostic value, mostly originating from animal solid peri-implant tissue studies [73,74,75]. However, in a recent human study, certain miRNA signatures appeared to be potential biomarkers for peri-implant conditions [76].

Nevertheless, it can be concluded that the microbiologic profile in peri-implantitis is complex and variable, with a prevalence of gram-negative anaerobic bacteria as well as other opportunistic microorganisms. Peri-implantitis sites comprise aggressive and antimicrobial-resistant microorganisms and exhibit a different microbial ecosystem compared to periodontitis sites [77]. Since a similar microflora composition has been found between healthy and diseased implant sites, the quantitative rather than the qualitative characteristics of the microbiota could be the key factor for the onset of peri-implantitis.

## 10. Treatment of Peri-Implant Diseases

### 10.1. Mechanical Decontamination of Implant Surface

The purpose of implant surface decontamination is to eliminate the adhered biofilm and to reduce the bacterial colonization to an extent that is compatible with peri-implant health [78,79]. The mechanical debridement may be performed using curettes, rotary titanium brushes, ultrasonic and air-abrasive devices [80,81,82,83,84]. Various types of curettes have been evaluated in the literature. Stainless steel curettes are contraindicated for implant surfaces according to several authors, due to the hardness of steel being greater than the titanium, potentially resulting in significantly more damage to the implant surface [78,85]. Titanium curettes have the same hardness of the implant surfaces, while carbon-fiber and Teflon curettes are softer; consequently, these curettes may be used to debride the implant surface without the risk of damaging it [86,87]. On the other hand, carbon-fiber and Teflon curettes have the drawback of breaking easily. While the general consensus in the literature seems to be that stainless steel curettes cause greater damage to the implant surface, titanium and plastic curettes have also been reported to roughen the implant surface, and not necessarily significantly less than the stainless steel ones [83,88].

Ultrasonic devices are also commonly used to debride the implant surface and they are as effective as the hand instruments in removal of the bacterial biofilm [80,83,85]. While regular ultrasonic tips are available, dedicated ultrasonic tips made of implant compatible materials have been proposed and used to treat the implant surface, the most prominent being carbon fiber, silicone or plastic ultrasonic tips [78]. Additionally, rotary titanium brushes are an option for mechanical decontamination of the implant surface with comparable results in the removal of debris to that found with ultrasonic tips [83].

Air-abrasive devices have been introduced in order to decontaminate the implant surfaces as well. However, these devices must be designed and used with caution in order not to damage the soft tissue or cause tissue emphysema when applied subgingivally [82,89]. The low-abrasive amino acid glycine powder has been demonstrated to be the most appropriate for subgingival decontamination [89]. In addition, air powder abrasion with glycine powder has been found to have a greater cleaning efficacy and it damages the implant surfaces less compared to ultrasonic devices or hand instruments [90].

### 10.2. Laser Implant Treatment and Combination Therapy

While it may suffice in the treatment of peri-implant mucositis and supportive peri-implant therapy, the exclusive use of mechanical therapy has not been demonstrated to be effective for re-osseointegration and the treatment of peri-implantitis [81,91,92]. Therefore, other procedures have been proposed such as the use of lasers alone, or adjunctive to the mechanical debridement. Many types of lasers have been described for this purpose including: erbium-doped yttrium aluminium garnet (Er:YAG); carbon dioxide (CO_2_); gallium aluminium arsenide (GaAlAs) diode; neodymium-doped yttrium aluminium garnet (Nd:YAG); erbium-and chromium-doped yttrium scandium gallium garnet (Er,Cr:YSGG) [93,94,95]. The decontamination capacity of these devices has been shown to be dose-dependent and, in high dose, each of those lasers was able to decontaminate the implant surface [95]. However, Er:YAG showed the greatest capacity of near-complete or complete bacterial decontamination over a wide range of power settings, while CO_2_ and GaAlAs diode lasers demonstrated less than optimal bacterial decontamination. The capacity of Nd:YAG laser to decontaminate the implant surface is still debated in the literature. The use of lasers alone has shown comparable results with air abrasive devices [96]. The combination of mechanical and laser treatment demonstrated a statistically significantly higher capacity of peri-implantitis resolution compared to mechanical treatment alone [97].

Mechanical therapies can also be combined with antimicrobial agents such as antiseptics and antibiotics [79]. The treatment should aim to effectively debride the implant, maintaining its surface’s integrity and not resulting in a favorable environment for bacteria. For the treatment of peri-implant mucositis, antimicrobial rinses were found to be beneficial as adjuncts to non-surgical mechanical treatment [98]. However, chlorhexidine in various forms has not shown any significant beneficial results as an adjunct to mechanical treatment of peri-implantitis [98,99,100].

In the non-surgical treatment of peri-implantitis, systemic and local antibiotics have been demonstrated to decrease BOP and PDs [98,101]. Systemic antibiotics as adjuncts to mechanical debridement had a significant positive impact on the treatment of modified implant surfaces, although adjunctive antibiotics did not affect the non-modified surfaces [100]. However, conflicting results have been reported on the adjunctive use of systemic antibiotics in the treatment of peri-implantitis. The combination of amoxicillin and metronidazole as an adjunct to non-surgical debridement for the treatment of peri-implantitis failed to demonstrate any additive benefit in microbiological and clinical results [102]. It has been concluded in a systematic review that the use of antibiotics in the treatment of peri-implantitis did not offer a long-term stable outcome; on the contrary, the use of systemic antibiotics could allow superinfection of the site by opportunistic microorganisms by creating a dysbiotic environment with a down-regulated local innate immune response [103]. At the same time, increasing the antibiotics resistance would make the site even more susceptible to peri-implantitis in the future.

Several studies showed positive results from the adjunctive use of local antibiotics [92,104]. Therapy of peri-implantitis by local delivery of tetracycline had a positive effect on clinical and microbiological parameters [105]. The beneficial use of repeated local minocycline microspheres as an adjunct to the mechanical treatment of peri-implantitis has also been studied in a randomized controlled clinical trial [106]. In this study, sites treated with local antibiotics demonstrated significant clinical improvements compared to control sites. The use of a simple non-surgical approach involving combined intrasulcular chlorhexidine irrigation and local delivery of minocycline hydrochloride has also demonstrated promising results in the treatment of infrabony defects around failing implants [107]. Nevertheless, overall, the use of systemic as well as local antibiotics is not sufficiently supported by the literature for the treatment of peri-implantitis, and more randomized controlled trials are required to warrant their adjunctive use [108,109,110,111,112,113]. Adjunctive use of local and systemic antibiotics in the treatment of peri-implantitis is shown in Table 2.

Similar results to adjunctive local antibiotics were achieved with the adjunctive use of photodynamic therapy to mechanical debridement [114,115]. While photodynamic antimicrobial treatment shows promise as an adjunct to mechanical treatment, the current level of evidence does not allow for meaningful conclusions [116]. Further clinical investigations regarding the clinical effectiveness of adjunctive photodynamic antimicrobial therapy are warranted.

The surface of the implant is a key factor that needs to be considered when selecting the type of combination therapy. Each implant surface has different properties and these implant surfaces may respond better to specific combined treatments [84]. Many chemical agents have been used as adjuncts to mechanical decontamination of the implant surface, hydrogen peroxide, phosphoric acid and ethylenediamine-tetraacetic acid (EDTA) being the most commonly reported in the literature [117]. However, whenever a chemical adjunct is utilized, it is important to maintain a pH ≥ 3; otherwise, noticeable morphological changes and corrosion on the implant surface occur and this may prevent re-osseointegration [118]. Implant surface decontamination methods, techniques and adjunctive chemical and antimicrobial agents are shown in Table 3.

### 10.3. Surgical Treatment Strategies for Peri-Implantitis

While non-surgical therapy may be a conservative treatment option, it has been shown that there is a high recurrence rate and often no resolution of the peri-implant disease [81,91,92,119]. Therefore, surgical therapy is often indicated for peri-implantitis treatment. The aims of surgical therapy are the decontamination of the implant surface, establishment of a positive hard and soft tissue peri-implant anatomy that promotes cleansibility and, when possible, regeneration of the infrabony defect [120]. Surgical approaches described for the treatment of peri-implantitis include: open-flap debridement (OFD), apically positioned flap (APF) and guided bone regeneration (GBR) [121,122,123].

**Figure 3:**
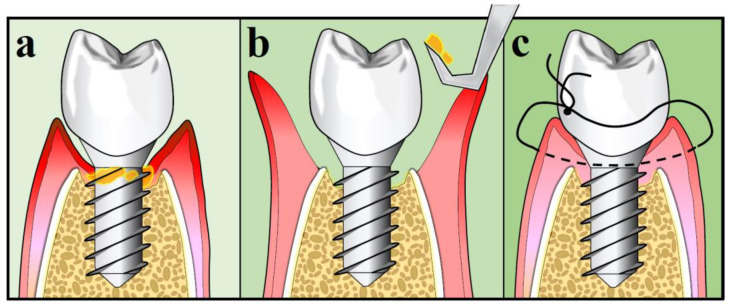
Open-flap debridement: (**a**) Pre-operative; (**b**) Intra-operative; (**c**) Flap closure.

In order to decontaminate the implant surfaces with OFD, a flap is elevated. Then, mechanical methods of decontamination with or without adjuncts are utilized, followed by repositioning of the flap at its original mucosal margin level [124]. It has been demonstrated that OFD is more effective than non-surgical treatment and may promote new osseointegration, especially on rough implant surfaces [91,120,125]. Adjunctive use of systemic azithromycin failed to demonstrate any additional clinical benefits compared to OFD debridement alone after one year [126]. The procedure of OFD is illustrated in Figure 3.

In addition to OFD, APF with or without bone re-contouring may often be indicated [127,128]. However, the success rate of this technique seems to depend on the initial amount RBL around the implants. If the RBL is <4 mm, a success rate of 74% has been reported, while in cases with RBL ≥4 mm, it dropped to 40% [128]. APF with and without bone re-contouring reported no further RBL long-term after the treatment in 78% of the cases [129]. Regarding the use of antibiotics as adjuncts to APF with osseous resection, a randomized controlled trial concluded that the benefits from the adjunctive use of antibiotics (amoxicillin) could not be sustained after three years [130].

**Figure 4:**
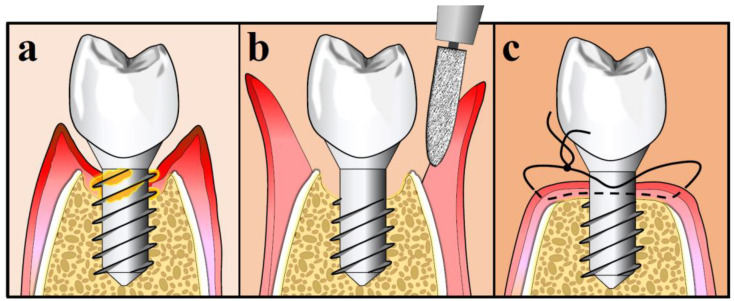
Apically position flap with implantoplasty: (**a**) Pre-operative; (**b**) Intra-operative; (**c**) Flap closure.

APF may also include implantoplasty, in an attempt to make the exposed implant threads smoother [127]. Implantoplasty was proven more successful than air-abrasive devices, titanium brushes, Er: YAG laser and chemical agents in removing biological debris from the implant surface [131]. Several concerns were raised on the mechanical and biological complications, such as destruction of the connection between the implant and the abutment, excessive heat generation and staining or inflammatory response to titanium particles deposition. However, a recent systematic review found no such adverse associations after short- to medium-time periods (≤36 months) [132]. However, it has been reported that the clinical results of APF were primarily influenced by the amount of RBL at the start point of the procedure, regardless of whether implantoplasty was performed or not [133]. The procedure of APF with implantoplasty is illustrated in Figure 4.

**Figure 5:**
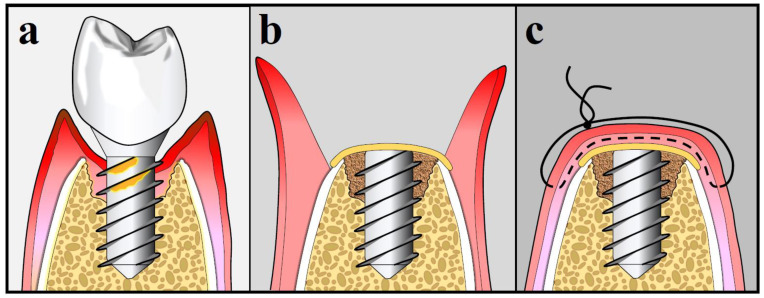
Guided bone regeneration: (**a**) Pre-operative; (**b**) Intra-operative; (**c**) Flap closure.

Osseous resective procedures have shown good results in peri-implantitis treatment. The success of osseous resective procedure depends on the characteristics of the infrabony defect (depth and shape). Regeneration procedures also may be attempted in selected cases. Evidence showed that in a bony defect of 3 or 4 walls and when the anatomy is favorable, guided bone regeneration (GBR) should be attempted as the first treatment of choice [134,135]. In addition, the FDI World Dental Federation consensus meeting on peri-implantitis stated that surgical osseous resective therapy has limited value long-term, and should be performed only in shallow infrabony defects where regenerative procedures are not recommended [123]. In published results of GBR used in the treatment of peri-implantitis, the type of implant surface may play a key role. Regeneration in non-modified implant surfaces demonstrated better results than in modified ones [129]. Furthermore, sand-blasted, large grit, acid-etched (SLA) implant surfaces responded better compared to titanium plasma-sprayed (TPS) implants [136]. As far as the GBR technique, a flap is raised to access the infrabony defect, decontamination of the implant surface is carried out with or without adjunctive antimicrobial agents and bone grafting material may be utilized to fill the defect with the subsequent addition of a barrier membrane [123,137,138,139,140,141,142]. Among the great variety of bone augmentation materials and membranes reported in the literature, no evidence suggests superiority of one over the others [123]. A recent randomized controlled clinical study evaluated the surgical treatment for infrabony peri-implantitis defects utilizing biodegradable prolonged release of local doxycycline formulated with β-tricalcium phosphate bone graft [143]. The results showed a statistically significant difference in clinical parameters between the test and the control groups at 12 months, supporting the use of adjunctive antibiotics in GBR procedures. As far as the selection of the antibiotic regimen after GBR for treatment of peri-implantitis, there have been multiple reports in the literature, but randomized controlled trials for the establishment of superiority of a specific protocol are warranted. The procedure of GBR is illustrated in Figure 5.

### 10.4. Success Rate of Surgical Peri-Implantitis Treatment

The effectiveness of the various types of peri-implantitis treatment is still under debate in the literature. However, with proper case selection and a strict maintenance protocol, surgical treatment appears to be the most effective in terms of resolution of peri-implantitis [122]. OFD procedures demonstrated a success rate of 47% for complete resolution of peri-implant inflammation at 1 year of follow-up [124].

Lagervall and co-workers obtained a success rate of 83% utilizing an APF with bone re-contouring in a retrospective study and a follow-up of 2–4 years [144]. Serino and colleagues reported a 74% success rate with APF and bone re-contouring at two years of follow-up in the treatment of implants with RBL < 5 mm [50]. Berglundh and co-workers, in a 2–11 years retrospective study of APF with and without bone re-contouring, reported a probability of no further bone loss of 77% with the absence of BOP and 83% of the sites exhibiting a PD < 5 mm [129]. Peri-implantitis treatment with GBR showed a 75% success rate at 2–4 years of follow-up and 53% at 5 years [144,145]. At 7 years, the success rate after treating peri-implantitis with GBR ranged from 14.3% to 58.3% depending on the implant surface [140].

## 11. Concluding Remarks

Peri-implant diseases are becoming increasingly prevalent, especially with the continuous rise in the use of dental implants. Several publications have been issued on peri-implant diseases, and while this manuscript does not provide any new information, it does provide the latest concepts and developments in the field and can serve as an overall guide for clinicians and surgeons. Peri-implant health, peri-implant mucositis and peri-implantitis were clearly defined at the 2017 World Workshop of the Classification of Periodontal and Peri-implant Diseases and Conditions. These universally accepted case definitions should be used in new epidemiological and clinical studies on peri-implant diseases for less heterogeneous results. Additionally, it is essential for clinicians to use the most updated definitions in their everyday practice, to come up with the correct diagnosis and treat the peri-implant condition accordingly. The most important risk factors for peri-implantitis are smoking, diabetes mellitus, history and presence of periodontitis and lack of proper maintenance. The iatrogenic factor of excess cement has also been strongly associated with the development of peri-implantitis around cementable restorations. With the diagnosis of peri-implantitis now clarified, it is imperative that a proper prognostication system is developed in the future. Peri-implantitis characteristics have been specified and defined on a clinical and radiographic level and the histologic and microbiological features were addressed in this paper. Peri-implant mucositis is predictably treated with non-surgical treatment, and this can be performed with various instruments, devices and antimicrobials. Regarding peri-implantitis, non-surgical treatment may not be as effective due to a higher recurrence rate and insufficient resolution of the disease. Peri-implantitis surgical treatment includes OFD, APF and GBR; however, case selection is critical to achieve a successful clinical result. While the reported results from already published clinical trials are encouraging, further randomized clinical investigations and properly controlled comparisons among the various surgical techniques need to be conducted in order for a predictable, evidence-based protocol to be established for the treatment of peri-implantitis.

## Figures and Tables

**Table 1:** Case definitions of peri-implant health and diseases according to the 2017 world workshop of the classification of periodontal and peri-implant diseases and conditions.

	Peri-Implant Health	Peri-Implant Mucositis	Peri-Implantitis (with Records)	Peri-Implantitis (No Records)
Visual signs of inflammation	-	+	+	+
BOP with/without suppuration	-	+	+	+
Increased PD vs. previous visit	-	-	+	≥6 mm
Increased RBL from initial remodeling	-	-	+ initial bone remodeling should not be ≥2 mm	≥3 mm apical to the most coronal part of the intraosseous implant portion

BOP: bleeding on probing, PD: probing depth, RBL: radiographic bone loss, (+): presence, (-): absence (adapted from [12]).

**Table 2:** Adjunctive use of local and systemic antibiotics in the treatment of peri-implantitis.

	Local Antibiotics	Systemic Antibiotics
Non-surgical debridement	Inconclusive results	Inconclusive results
Open Flap Debridement	No evidence	No benefit
Apically-Positioned Flap	No evidence	No benefit
Guided Bone Regeneration	Encouraging short-term results	Recommended

**Table 3:** Methods, techniques, adjunctive and antimicrobial agents for implant surface decontamination.

Method of Decontamination	Advantages	Disadvantages
Stainless Steel Curettes	• Good debris removal	• Significant implant surface alteration
Titanium Curettes	• Good debris removal	• Minimal implant surface alteration
Plastic Curettes	• No implant surface alteration	• Ineffective debris removal • Fragile
Ultrasonic with Dedicated Tip	• Excellent debris removal	• Minimal implant surface alteration
Ultrasonic without Dedicated Tip	• Total debris removal	• Significant implant surface alteration
Titanium Brushes	• Excellent debris removal	• Significant implant surface alteration • Fragile
Air-abrasive Devices	• Excellent debris removal • Minimal implant surface alteration	• Soft tissue damage with inappropriate use
Lasers	• Excellent bacterial decontamination	• Dose-dependent efficacy
Chlorhexidine	• None	• No adjunctive effects
Chemical Agents (H_2_O_2,_ H_3_PO_4,_ EDTA, etc.)	• Controversial	• Morphologic agents and corrosion with pH < 3
Systemic Antibiotics	• Limited evidence	
Local Antibiotics	• Limited evidence	
Photodynamic antimicrobial	• Limited evidence	

H_2_O_2_: hydrogen peroxide, H_3_PO_4_: phosphoric acid, EDTA: ethylenediamine-tetraacetic acid (adapted from [78,83]).
